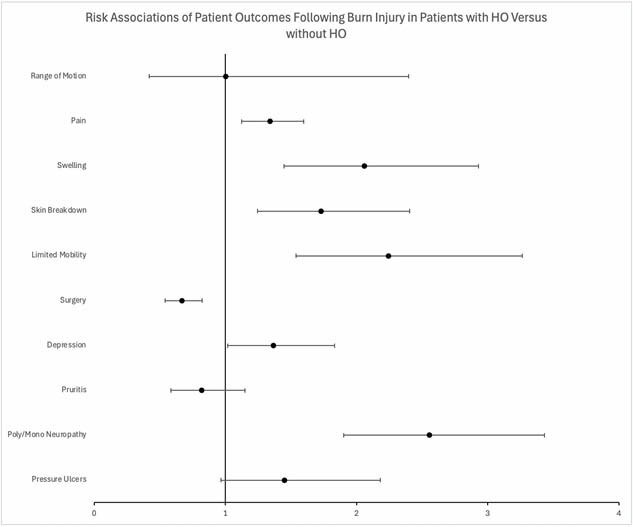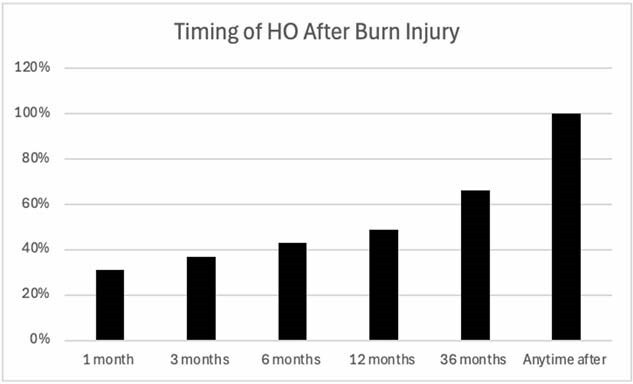# 885 Heterotopic Ossification After Burn Injury Results in Worse Outcomes

**DOI:** 10.1093/jbcr/iraf019.416

**Published:** 2025-04-01

**Authors:** Jasmine Chaij, Mbinui Ghogomu, Farhad Marzook, George Golovko, Juquan Song, Steven Wolf, Amina El Ayadi

**Affiliations:** University of Texas Medical Branch; University of Texas Medical Branch; University of Texas Medical Branch; University of Texas Medical Branch; University of Texas Medical Branch; University of Texas Medical Branch; University of Texas Medical Branch

## Abstract

**Introduction:**

Heterotopic ossification (HO) is the formation of extra-skeletal bone within soft tissue that occurs after injuries like severe burns. Development of HO complicates healing after injury. We aimed to elucidate factors influencing the development of HO in burn survivors and effects on recovery.

**Methods:**

Using the TriNetX database, a large, federated research network of de-identified patient data, we compared burned patients who developed HO after injury and those who did not. Cohorts were propensity-matched by age, gender, race, ethnicity, and % total body surface area (TBSA) burned. We then evaluated incidences of outcomes after injury among both cohorts.

**Results:**

Of the population, 0.12% developed HO after injury with most identified greater than 6 months from injury. HO was more common in those with head, upper extremity, or lower extremity burn involvement (p< 0.0001). The HO group was older (48.1±19.5 yrs. vs 32.5±22.8 yrs., p< 0.001), and male (60.3% vs 51.1%, p< 0.001). African American patients were more likely to develop HO (22.2% vs 17.5%, p< 0.001). Additionally, those with HO exhibited a more than doubled risk of neuropathy, limited joint mobility, and documented oedema than those who did not. Additionally, risk of skin breakdown, pain, and depression was higher in those who developed HO while additional reconstructive operations was interestingly lower.

**Conclusions:**

Burn survivors more likely to develop HO are older, male, greater injury severity, and of African-American ethnicity. Identification of HO was more likely greater than 6 months from injury. After propensity matching for burn severity and demographic factors, those with HO have comparatively poorer outcomes.

**Applicability of Research to Practice:**

This study shows that burn patients who develop heterotopic ossification (HO) have worse functional outcomes than those without HO. Early identification and intervention can reduce complications, emphasizing the need for screening and proactive management of HO in burn care protocols.

**Funding for the Study:**

Database funding by National Center for Advancing Translational Sciences.